# Unwanted isolation: An obstacle to constructive interaction between oncology nurses and their patients

**DOI:** 10.1002/nop2.882

**Published:** 2021-04-04

**Authors:** Amir Hossain Pishgooie, Jamileh Mohtashami, Foroozan Atashzadeh‐Shoorideh, Neda Sanaie, Ensieh Fathollahzadeh, Victoria Skerrett

**Affiliations:** ^1^ Department of Medical‐Surgical Nursing AJA University of Medical Sciences Tehran Iran; ^2^ Department of Psychiatric Nursing and Management School of Nursing and Midwifery Shahid Beheshti University of Medical Sciences Tehran Iran; ^3^ Department of Psychiatric Nursing and Management School of Nursing & Midwifery Shahid Beheshti University of Medical Sciences Tehran Iran; ^4^ Student Research Committee School of Nursing & Midwifery Shahid Beheshti University of Medical Sciences Tehran Iran; ^5^ Flinders University College of Nursing and Health Sciences Adelide SA Australia; ^6^ Birmingham City University Birmingham UK

**Keywords:** Cancer, Communication, Interaction, Nurse, Patient

## Abstract

**Aim:**

The current study was carried out to investigate the obstacles faced by oncology nurses in their interactions with their patients.

**Design:**

This research is a descriptive qualitative study.

**Methods:**

In this study, conventional content analysis was used for analysing the data collected from 26 oncology nurses. The participants were selected through purposive sampling. Semi‐structured interviews were used for collecting data. Data analysis was conducted with Elo and Kyngäs's approach.

**Results:**

The results included three categories: “role conflict,” “role overload” and “inefficient interaction,” and 10 subcategories.

## 1 INTRODUCTION

Using effective communication skills is an integral part of nursing profession and is regarded as an important element in nursing care and nursing education (Ardalan et al., 2018). Weak relationships with patients may hinder the process of gaining important information about them, possibly resulting in misinterpretation of patients’ information and causing distrust between patients and healthcare providers (Dean & Street Jr, 2014). It is essential to establish suitable communication with cancer patients so that they can be involved in decision‐making about their care and helping professionals to alleviate the side effects of cancer treatment (Treiman et al., 2018). In most of the cases, cancer patients feel they do not have enough information about cancer, which can lead to their uncertainty, anxiety and depression (Martin et al., 2019; Zwingmann et al., 2017).

The use of good communication skills in constructive interactions plays an important role in fulfilling health providers and patients’ satisfaction. It also increases the likelihood of patient adherence to treatment instructions, better decision‐making and positive healthcare outcomes for patients (Araki, 2019). Therefore, recognizing the obstacles to constructive interaction between oncology nurses and patients plays an important role in the proper formation of therapeutic communication.

## 2 BACKGROUND

Through better interactions, especially by considering patients’ feelings and symptoms, nurses can provide more effective and efficient health care (Hultstrand Ahlin et al., 2019). Empathetic communication between nurses and patients results in better understanding, psychological support, improvement in patients’ physical, mental and behavioural conditions, and patients’ increased comfort (Bauchat et al., 2016). A good rapport with patients can boost cooperation, and increase motivation, helping nurses to reduce the costs of achieving organizational goals (Treiman et al., 2018).

In Arkorful's study, barriers to effective therapeutic communication between nurses and patients include differences in background, conflict in the patient–nurse relationship and language barrier. Other identified nurse‐related barriers include human resource challenges, patients' lack of trust in nurses’ competency, interference from patients’ family and friends, inadequate knowledge on the part of the nurse, patient's dissatisfaction with progress and patients’ emotional fluctuations (Arkorful et al., 2020).

Through good interactions, nurses become aware of patient's needs and can properly respond to them. Although good interaction between nurses and patients is a crucial and effective prerequisite for successful health care, it has not been thoroughly investigated yet, especially in patients with cancer (Fakhr‐Movahedi et al., 2016). Effective interaction between nurses and patients leads to positive outcomes such as reduction in stress and feelings of guilt, more tolerable disease symptoms, increase in patients’ satisfaction, acceptance of and adaptation to their disease, improvement in their physiological and performance conditions (Ardalan et al., 2018). It seems that it should be investigated with reference to nurses’ thoughts, feelings and personal experiences. This study provides an in‐depth investigation into nurses’ perspectives on obstacles to constructive interaction between nurses and patients. In this way, barriers to therapeutic communication between the patient and the nurse can be minimized and eliminated over time. The aim was to explore nurses’ experiences and perception obstacles to constructive interaction between oncology nurses and their patients.

## 3 METHODS

### 3.1 Setting

This research was conducted where the phenomenon occurred. Five educational hospitals in Tehran (Capital of Iran) were selected. These hospitals provide care and treatment services to oncology patients. All patients were in the active phase of the disease and underwent chemotherapy between one month and fifteen months after diagnosis. All patients were adults, and their mean age was 49.21 ± 6.32.

### 3.2 Design

In this research, the method of qualitative content analysis with a conventional method was used. This approach includes identifying, interpreting and conceptualizing meaning units of qualitative data, which lead to the formation of valid data with the aim of creating new insights and knowledge about the field under study (Krippendorff, 2018).

### 3.3 Participants

In this qualitative study, 26 oncology nurses from educational hospitals in Tehran were selected by purposive sampling. A qualitative study is conducted with the aim of obtaining the most information about the phenomenon and therefore the selection of participants with a variety of demographic information, work experiences to help the researcher achieve this goal (Palinkas et al., 2015). Attempts were made to have a wide range of participants in terms of age, gender, academic degree and work experience in oncology ward.

### 3.4 Data collection

The study was conducted in 2018 and 2019. After explaining the purpose of the research to the participants, semi‐structured individual interviews were conducted (Krippendorff, 2018). Interviews began with general questions such as “Can you talk about your communication experiences with cancer patients?” Follow‐up questions were asked based on what the participants disclosed. The interviews were conducted individually and in a private setting selected by the participants in hospital and lasted 30 to 55 min, and the average duration was 35 min. At the first opportunity after the interview, they were transcribed and data were collected and analysed simultaneously until data saturation was reached. The data saturation was reached after 23 interviews, and four participants took part in a follow‐up interview. Data and coded interviews were shared with the four participating at different sessions, and their opinions were sought, and the results of the study were given to four participating to judge on the similarity of the results with their own experiences.

### 3.5 Data Analysis

The three‐step approach proposed by Elo and Kyngäs (Elo & Kyngäs, 2008) was used to analyse the data. During the preparation phase, all interviews were transcribed, and following that, the interviews, as units of analysis, were analysed three times. In the organizing phase, after reviewing the semantic units related to the research questions, the initial codes were extracted through the inductive approach. In the reporting phase, the semantically similar initial codes were placed in their respective subcategories. Subsequently, the resulting subcategories were reviewed, and after repeated comparisons, the same subcategories merged to form the main categories.

### 3.6 Trustworthiness

Lincoln and Guba's criteria were used to examine the trustworthiness of data (Lincoln & Guba, 2017). Researchers maintained contact with the participants and took considerable time to check the results of data analysis with them. The researchers and two expert faculty members who had already conducted several qualitative studies checked the analysed data. Attempts were made to collect data from a wide range of participants in terms of their gender, age, academic degree and length of hospitalization. Finally, to make sure confirmability was achieved, all research steps were documented and reviewed.

### 3.7 Ethical Considerations

The study was approved by the ethics committee of Shahid Beheshti University of Medical Sciences (IR.SBMU.RETECH.REC.1396.552). Additionally, informed consent was obtained from the participants to conduct interviews, record their voice and use the data.

## 4 RESULTS

The average age of participants was a 33.27 ± 6.87 year, 70% were female, 85% had BSc degree in Nursing. Participants’ information is described in detail in Table 1. The results of the final analysis were collated in the form of 10 subcategories, 3 categories (Table 2). Nurses' comments are expressed using direct quotations.

**TABLE 1:** Participants’ characteristics

Participants’ characteristics	*N* (%)
Gender	
Men	8 (30)
Women	18 (70)
Education	
BS degree holders	22(85)
MS degree holders	4 (15)
Age (Mean± *SD*)	33.27 ± 6.87
Work experience in oncology ward (Mean± *SD*)	6.09 ± 2.25

Abbreviations: %, Per cent; *N*, Number; *SD*, Standard Deviation.

**TABLE 2:** Overview of the categories and subcategories

Categories	Subcategories
Role conflict	‐ Large number of roles ‐ Role interference ‐ Conflicting expectations
Role overload	‐ Administrative bureaucracy ‐ Shortage of manpower/large number of patients
Inefficient interaction	‐ Cultural differences ‐ Isolation ‐ Misunderstanding ‐ Bad temper ‐ Indifference

### 4.1 First category: Role conflict

Role conflict was one of the categories which emerged in data analysis. Oncology nurses experienced role conflict due to numerous responsibilities they had, conflict between these responsibilities and conflicting expectations. In fact, they argued that the healthcare workers had to fulfil many different roles simultaneously, and interference of these roles with each other hindered them from properly fulfilling their responsibilities. This category consists of three subcategories.

“Yesterday, a patient was talking to me about his problems. Suddenly someone summoned me from the station and asked me to do something. Well, I don't know what I should do in this situation. I somehow apologized to the patient, but I noticed that he became upset. I had to leave him alone when he was talking” **(Large number of roles)**.

Some other participants said that they experienced role conflict when the expectations of a particular responsibility hindered them from fulfilling the expectations of another one. This was especially true when the participant had some expectations of that responsibility, while others expected him/her to do something else in that capacity. Thus, they were not able to establish a suitable relationship with the role and were not willing to fulfil their relevant duties.

“There were times when the patient was talking to me but it was the end of the shift and I had to go get my son from school. Well, on the one hand, I am a mother and I have to play the role of a mother well, and on the other hand, I am a nurse and I have to pay attention to the wishes and needs of patients” (Role interference).

“The patients also expect that we always be next to their bedside and do not leave them alone. However, we have other patients too, and we should take care of them as well. All of them have their own expectations. Sometimes I don't have enough time to take care of all of them. Therefore, I should pay a quick visit and cannot allocate further time” (Conflicting expectations).

Due to the conflict that was observed in such cases, this category was named "role conflict," which, according to what the participants said, is a hindrance to allocate enough time to interact with patients.

### 4.2 Second category: Role overload

Role overload was another category emerging form the data. It entailed two subcategories.

The participants believed that they had to devote a large amount of their work time to administrative bureaucracy, which meant they would spend less time interacting with patients.

“We didn't have this large amount of paperwork in the past. They ask us to do a lot of paperwork these days. For example, I should do the accreditation too. I should also attend relevant classes and document everything. All of these requires a lot of time. Sometimes I don't have any free time to talk to patients” (Role overload).

Some of participants stated that the nurse to patient ratio was not adequate and that the healthcare workers were overstretched and hence had no time to attend to patients.

“Everybody knows that we have a small number of nurses in hospitals and the situation is below the standard level. Nurses should take care of many things. I have frequently been summoned while talking to patients. I cannot attend to other things that I should do if I want to spend time with patients” (Shortage of staff members/large number of patients).

### 4.3 Third category: Inefficient interaction

This category comprised five subcategories, including cultural differences, isolation, misunderstanding, bad temper and indifference. Participants mentioned these subcategories, which have to do with the challenge in the relation between healthcare workers and patients.

Regarding cultural differences, it was found that sometimes discrepancy in the customs of a particular ethnic group would hinder healthcare workers and patients to have a constructive interaction.

“Many times, the cultural differences of patients with us have prevented the formation of a proper relationship. I had a patient who, due to cultural issues, did not like to be called by her first name. I did not know this. When I called her by her first name, she was upset and did not talk to me anymore.” (Cultural differences).

On the other hand, due to their pain and suffering, sometimes cancer patients are not in mood of communicating with others and would like to be left alone. In such cases, they prefer isolation and solitude.

“Sometimes when I proceed to patients’ bedside, I notice they are not in mood of talking. Therefore, I respect their and leave them alone. I will talk to them later” (Isolation).

The participants believed sometimes attempting to build communication with patients could backfire and might lead to misunderstanding. In such cases, patients are less willing to communicate with healthcare workers in subsequent referrals.

“I tried to talk to a patient to help him, but he thought I was feeling pity for him because of his cancer. He thought I was trying to talk to him because he was dying. Therefore, he was not willing to talk to me” (Misunderstanding).

Bad temper was another subcategory emerging from what participants said. According to interview data, it was observed in both patients’ and healthcare workers’ behaviour and was a hindrance to constructive interaction between them.

“Some of patients are very bad‐tempered. I am a nurse and have questions. They lose their temper as soon as you ask a couple of questions. So talking comfortably with them is impossible” (Bad temper).

“Yesterday, one of my patients told me: I had a lot of pain once and asked for painkiller. It took a lot of time before I received an injection. None of nurses do not pay attention to my pain and have no idea about my sufferings caused by this disease” (Indifference).

Based on the emerged categories and subcategories, it seems that conflict and role overload prevent healthcare workers from having enough free time and establishing suitable communication with patients. On the other hand, sometimes the interaction is not efficient enough, which will have negative consequences for patients or healthcare workers. After frequently reviewing what the nurses said and examining the emerged categories, it seems that the patients eventually fell into unwanted isolation, which could have been prevented. In fact, when the patients’ and healthcare workers’ attempts to interact constructively with each other fail, patients fall into unwanted isolation and fail to experience a constructive interaction. It can be said that obstacles to constructive interaction between oncology nurses and patients lead to unwanted isolation of the patient.

## 5 DISCUSSION

The current study was done to explain the interaction experiences oncology nurses with their cancer patients. The findings revealed that obstacles to constructive interaction between oncology nurses and their patients could be attributed to role conflict, role overload and insufficient interaction. Loneliness is a painful experience for patients and lack of proper communication with the nurse can lead to isolation in patients (Karhe & Kaunonen, 2015).

Role conflict comprised the large number of roles, role interference and conflicting expectations which members of staff experience. Conflict refers to a situation in which two or more perceived roles in a work environment are not compatible; hence, it would be difficult for individuals to adapt themselves to these roles. Lack of information and clear job descriptions in the work environment prevent individuals from properly fulfil their duties and damages interpersonal interactions, role conflict leads to disappointment and is the cause of instrumental relationship between patients and the nurses (Pishgooie et al., 2016).

Naturally, instrumental relationships do not include emotions, which constitute the basis for expressive relationships. Nurses have authority in communicating with patients. The interaction between nurses and patients plays a social role in reducing the damaging risks of disease in society. When it comes to chronic illness, various effects of disease and psychosomatic disorders should be taken into account in the relationship between nurses and patients (Heidarnia & Heidarnia, 2016). The trust established between the two parties plays a statistically significant role in building effective relationship between patients and the oncology nurses (Hong & Oh, 2019).

Some of the participants believed that administrative bureaucracy and shortage of staff, which are the subcategories of role overload, should be blamed for hindering constructive interaction. In this regard, Cox et al., (2014) argue that the shortage of nurse is a growing trend across the globe. They mentioned various reasons for this attrition including lack of job satisfaction profession to care for ageing parents, demographic changes in patients, inappropriate recruitment, economic conditions, inappropriate training of nurses and the supervision procedure (Cox et al., 2014). In addition, bureaucracy is an indispensable part of all societies and big organizations; it is a complicated and dynamic process which stems from frequent and time‐consuming use of regulations and working procedures (Andrews et al., 2017). Role overload is a major reason for job stress, which in turn originates from factors like lack of enough time to do the assigned duties, the existence of very high standards that are not compatible with the individual's skills and abilities, and assignment of duties and responsibilities that are beyond the individual's capabilities (Kimura et al., 2018; Zhang et al., 2019).

Role overload and role conflict are the main stress factors that put pressure on individuals and reduce their energy levels, thus weakening their job performance, creativity and organizational behaviour (Montani & Dagenais‐Desmarais, 2018). Reduction in people's energy levels, in turn, may lead to lack of cognitive and emotional balance and mistakes (Zhang et al., 2019). As a major professional stress factor, the role overload is a main reason for individuals’ mental disorder (Kimura et al., 2018), which hinders constructive interaction. Shortage of staff and role overload lead to reduction for interaction, which in turn results in patients’ dissatisfaction and decline of the provided healthcare quality (Atashzadeh Shoorideh et al., 2012).

Inefficient interaction is the last category emerging in this study. The participants argued that cultural differences, isolation, misunderstanding, bad temper and indifference would lead to inefficient interaction. This type of interaction can cause chronic stress, dissatisfaction and mistrust for patients (Banerjee et al., 2016).

It seems that various personal and organizational factors influence the interactions between patients and the nurses (Pazargadi et al., 2015). Effective nurse–patient interaction leads to trust, calmness, safety, meaningful relationship, self‐esteem, multidimensional well‐being and prevention/reduction of anxiety and depression (Präg et al., 2017). The quality of the relationship between nurse and patient is a potential source of hope for patients. Through attending training programs, nurses should learn how to proceed to patients’ bedside, gain enough knowledge about patients without any prejudice, actively listen to patients, consciously choose words that will enhance the interaction quality and become familiar with theoretical perspectives about hope, genuine and empathetic nurse–patient interaction, self‐respect and human dignity (Haugan et al., 2016).

One of the advantages of establishing good relationships with cancer patients is their consequent increased satisfaction with the provided nursing care (Prip et al., 2018; Treiman et al., 2018). The findings of the study carried out by Kim‐Soon et al. indicate that cancer patients are happy with the interaction they have with their nurses. However, another study has demonstrated that, while interacting with cancer patients, nurses do not facilitate the relationship. Indeed, they mainly opt for a closed and vague interaction, which leads to many problems for patients (Kim‐Soon et al., 2017). Finally, nurse should encourage and support patients through informal and transparent interactions so that they will be involved in the treatment process (Reeve et al., 2017). Therefore, it is necessary to establish effective interaction between the oncology nurses and patients so that their needs and demands will be identified.

### 5.1 Limitation

This research has some limitations. Given that the participants of the present were all oncology nurses, the reasons for the lack of effective communication between nurse and patient from the perspective of cancer patients have not been investigated.

## 6 CONCLUSION

The results of this study showed that role conflict, role overload and inefficient interaction led to unwanted isolation, which hinders constructive interaction between the nurses and their patients. Given that interaction is a major factor in providing healthcare services, authorities and managers should take necessary steps to eliminate the obstacles to such interaction. Given that interaction is a major factor in providing healthcare services, authorities and managers should take necessary steps to eliminate the obstacles to such interaction. The findings of the current research can be the basis for further research on the factors related to the other communication patterns of team treatment with cancer patients.

## CONFLICT OF INTEREST

The authors have no funding or conflicts of interest to disclose.

## AUTHORS' CONTRIBUTIONS

All authors (A‐HP, JM, FA‐SH, EF, VS and NS): Participation in the conception and design of the study. A‐HP and FA‐SH: Data collection and preparation of the first draft of the manuscript. JM, EF, VS and NS: Revision and checking the proposal, the analysis and interpretation of the data and design the article. A‐HP and FA‐SH: Analysis and interpretation of the data and drafting the manuscript. FA‐SH, EF and NS: Manuscript revision. VS: Manuscript translation. All authors read and approved the final manuscript.

## Data Availability

The data that support the findings of this study are available on request from the corresponding author. The data are not publicly available due to privacy or ethical restrictions.
